# The Pre-Exposure Prophylaxis Stigma Scale: Measurement Properties of an Adaptation in German and French

**DOI:** 10.3389/ijph.2024.1606658

**Published:** 2024-04-29

**Authors:** Alexander Ort, Tess Bardy

**Affiliations:** Faculty of Health Sciences and Medicine, University of Luzern, Luzern, Switzerland

**Keywords:** pre-exposure prophylaxis, scale validation, stigma and discrimination, MSM, HIV and AIDS

## Abstract

**Objectives:**

This study aimed to adapt and validate the HIV PrEP Stigma Scale (HPSS) in French and German languages (HPSS-FR/DE) and assess its applicability across diverse linguistic and cultural contexts.

**Methods:**

The original scale was adapted to French and German and administered through an online survey in multiple European nations. A four-factor structure was extracted from the data, including negative social consequences, social pressure, self-support, and external support. The scale’s construct validity, reliability, and cross-linguistic consistency were evaluated.

**Results:**

The adapted HPSS-FR/DE demonstrated robust psychometric properties, good construct validity, acceptable reliability, and consistent measurement across different languages. This adaptation enhances its utility in multicultural settings, offering a comprehensive tool to assess PrEP-related stigma.

**Conclusion:**

This study provides a suitable tool to address PrEP stigma in multicultural environments to enhance PrEP uptake and adherence among men who have sex with men. Moreover, it lays the groundwork for further investigations into PrEP stigma across diverse populations and cultural settings, enabling the development of targeted public health interventions and policies to combat this issue effectively.

## Introduction

The ongoing global HIV pandemic continues to be a significant public health concern, with millions of new infections reported annually [1]. Despite various evidence-based behavioral HIV prevention interventions, the number of new HIV infections remains alarmingly high, particularly among men who have sex with men (MSM), who face not only physical health challenges but also the burden of stigma and exclusion due to their infection [2].

In addition to health promotion efforts that seek to reduce stigma and encourage healthy behavior, recent biomedical innovations, such as pre-exposure prophylaxis (PrEP), have the potential to play a crucial role in curbing the epidemic [3, 4]. PrEP, an antiretroviral drug taken orally in a specific regimen, has been shown to reduce the risk of HIV infection by up to 92 percent when taken adherently, making it an effective tool for protecting individuals from contracting the virus [5–7]. Moreover, given the ongoing social exclusion and stigmatization faced by people living with HIV, PrEP has been recognized as not only a physical health preventive measure but also a tool to safeguard the mental health of high-risk groups, providing increased control and safety regarding sexual health and reducing stigma at both individual and social levels [8].

Although PrEP is highly effective and offers substantial benefits, its adoption remains notably low in certain populations, regions, or countries where its use would be most beneficial. This selective underutilization underscores a significant missed opportunity to reach its full preventive potential and can be attributed to various factors, including lack of awareness, low perceived risk of HIV acquisition, cost barriers, and the inconvenience of required follow-up visits [9].

One crucial factor that influences the uptake, adherence, and discontinuation of PrEP is the presence of PrEP shaming, wherein individuals who choose to use PrEP as a preventive measure experience stigmatization and negative attitudes from various sources, including friends, family, sexual partners, and even their own communities [10]. PrEP shaming can take different forms, such as slut-shaming, moral judgment, misconceptions about the drug, or associating PrEP use with promiscuity or irresponsibility [9, 11].

The impact of PrEP shaming on people’s attitude toward the drug and their behavior to utilize it as a prevention tool is severe [12]. Stigmatization can adversely affect individuals’ willingness to seek information or access the medication, leading to decreased uptake among those who could benefit from it. Moreover, PrEP shaming can impair mental health through induced feelings of guilt or shame [9], undermining individuals’ commitment to adhering to the prescribed regimen, which is crucial for the effectiveness of PrEP (see above). Additionally, PrEP shaming can ultimately contribute to the discontinuation of PrEP use, as individuals facing social pressure or self-doubt may decide to cease medication intake, leaving them vulnerable to HIV infection [13–15]. The adverse effects of PrEP shaming highlight the urgent need to address the issue to ensure effective HIV prevention and safeguard the mental health of at-risk populations.

Given the wide and seemingly still frequent appearance of shaming in various settings and its significant implications for the success of PrEP as a method of HIV prevention, it is of utmost importance to broaden our understanding of PrEP stigma. One crucial element in achieving this goal is the availability of instruments to assess PrEP stigma. While a couple of instruments have been introduced [16–18], limiting factors hinder a holistic understanding of PrEP stigma on a global and multicultural level. First, the diverse use of different instruments across studies presents a challenge in synthesizing findings and comparing results (for an exemplary overview, see [12]). The absence of standardized measurement tools hinders the establishment of a comprehensive and consistent understanding of PrEP stigma and its global implications. Existing studies largely focus on English-speaking or Anglo-American contexts [11, 19, 20] or specific linguistic communities within these settings, relying primarily on qualitative approaches [11, 14, 20–23]. While such community-specific efforts are essential for gaining an in-depth understanding of the issue’s occurrence, particulars, and consequences, the findings available to date lack generalizability and do not enable account for diverse cultural and linguistic backgrounds. To foster a more inclusive and nuanced comprehension of PrEP stigma, it is imperative to investigate it on a broader scale, encompassing multiple languages and diverse cultural contexts. Adopting standardized measurement tools and expanding research efforts can enhance our understanding of PrEP stigma’s impact across various communities and facilitate the development of targeted interventions and policies to address this issue effectively.

This research endeavors to overcome these limitations in our comprehension of PrEP stigma by adapting an existing scale, namely, the HIV PrEP Stigma Scale (HPSS) developed by Siegler et al. [18], for French and German language contexts. The scale will be tested following the translation and adaptation process to validate its effectiveness and applicability across diverse linguistic and cultural contexts. The overarching goal is to contribute to a broader comprehension of PrEP stigma and create a more comprehensive and culturally sensitive framework for its assessment and mitigation on a multicultural and (prospective) global scale.

Furthermore, the validated measures will empower future research to understand PrEP stigma at a country-specific level and enable cross-cultural comparisons. Furthermore, health promotion and disease prevention experts can utilize these results to design targeted interventions to address and overcome PrEP stigma [24]. As PrEP is used in over 100 countries worldwide [25], adapting the scale for use in multiple linguistic settings is a critical step toward its broader implementation in the fight against HIV. Finally, in extending our understanding of the magnitude and role of PrEP stigma, this study will also contribute to meeting a recent call in Nature (Bose, 2022) for behavioral sciences to consider lessons learned from biomedical preventive measures, like PrEP, to promote the uptake of forthcoming HIV vaccinations.

## Methods

### Adaptation of the HIV PrEP Stigma Scale (HPSS)

Developed and validated by Siegler et al. (2020), the HIV PrEP Stigma Scale (HPSS) is a 13-item survey instrument “[…] based on a stigma framework with three domains (internal, anticipated, and experienced stigma) and on three attributes abstracted from PrEP stigma literature (shame, character judgments, social support)” [18]. Each item evaluates self-reported anticipated or experienced stigma among MSM, reflecting MSM’ barriers to PrEP adoption, on a five-point Likert scale, from 1 “Strongly agree,” to 5 “Strongly disagree.” Higher scores indicate higher anticipated or experienced stigma and, thus, higher barriers to taking PrEP.

In this study, the HPSS will be applied in the context of experienced stigma, i.e., for MSM who are taking PrEP or have been taking it before. Minor adaptations were carried out to fit the wording of the scale for these two populations. It was then translated from English (US/GB) to French (FR) and German (DE) following the guidelines from Epstein et al. (2015) to create the HPSS-EN/FR/DE. Translations were carried out for each language by native speakers, compared, and refined to create the final version of the instrument in each of the two additional languages. For an overview, see Supplementary Table SA1.

### Data Collection and Participants

The translated HPSS scale (HPSS-EN/FR/DE), following appropriate adaptations, was incorporated into an online survey that was dispensed across four European countries: United Kingdom, France, Switzerland, and Germany over a span extending from December 2020 to April 2021. The survey was accessible in three languages: English, German, and French. Importantly, involvement in the survey was on a voluntary basis and hinged upon the condition that each participant explicitly furnished their well-informed and unambiguous consent before participation. The survey assessed a comprehensive range of demographic characteristics, as well as an assessment of participants’ knowledge, experience, and utilization of PrEP. The final dataset comprises 802 observations, forming the basis for subsequent analysis. The study has been conducted in adherence to prevailing ethical norms. An ethical waiver was obtained from the institutional review board, and the study adheres to widely recognized ethical guidelines for research involving human subjects.

MSM were recruited using various methods. Snowball sampling was primarily employed, utilizing mailing lists from various AIDS and MSM-specific associations to reach potential participants. Counseling and testing centers in the four countries also supported the recruitment process of the study by distributing posters and flyers. The study was also shared on social media platforms (i.e., Facebook, Instagram) of various LGBTQ+ and HIV/AIDS activists.

### Statistical Procedure

A single Exploratory Factor Analysis (EFA) with promax rotation was performed to ascertain the optimal structure of the adapted measurement tool. The EFA was conducted on a random selection of half the sample (*n* = 401). The determination of the number of factors to extract was guided by scree plot analysis, parallel analysis, and the conceptual coherence of the rotated factors. Notably, factor loadings equal to or exceeding 0.3 were deemed indicative of robust associations [26]. Subsequently, a Confirmatory Factor Analysis (CFA) was conducted using the other half of the sample (*n* = 401) to verify the factor structure. Diverse indicators were employed to evaluate the adequacy of fit within the extracted factor model. These indicators included the Root Mean Squared Error of Approximation (*RMSEA*), the Tucker-Lewis Index (*TLI*), the Comparative Fit Index (*CFI*), and the Standardized Root Mean Squared Residual (*SRMR*). Thresholds for a reasonable model fit were set to: *RMSEA* ≤ 0.05, *CFI* > 0.9, *TLI* > 0.9, and *SRMR* ≤ 0.05 [27, 28]. Finally, a multigroup confirmatory factor analysis (MGCFA) was performed on the total sample (*n* = 802) to test whether the adapted instrument elicits similar response patterns across languages, i.e., ensuring that it is measuring the same construct across the different translations, using configural invariance (model 1), metric invariance (model 2), and scalar invariance (model 3). Model 1 tests that the factor structure is invariant across groups. Model 2 holds factor loadings equal across groups. Model 3 further constrains the item-intercepts. Because *RMSEA* and *SRMRA* are affected by smaller sample sizes of the English, German, and French language sub-groups for analysis [29], the thresholds for reasonable fit for those analyses were set to *RMSEA* ≤ 0.08 and SRMR ≤ 0.08. Measurement invariance was assessed via changes (Δ) in the goodness of fit with Δ*CFI* ≥ −0 .005, Δ*RSMEA* ≤ 0.010 or a Δ*SRMR* ≤ 0.025 (≤0.005 for intercept invariance) indicating invariance [30]. General reliability of the validated structure was performed overall and for English, French, and German using Cronbach’s alpha and McDonald’s omega, with α and ω > 0.70 reflecting acceptable internal consistency.

## Results

### Sample Description and HPSS-EN/FR/DE Scores

Table 1 displays the sample characteristics. The final sample comprises 802 MSM individuals aged 18–76 (*M* = 30.40, *SD* = 9.95). About 88% of the sample currently takes PrEP and over half of the sample reported having obtained a higher education degree. Regarding dating and sex life, most individuals indicated having sexual contact without being in a relationship (41%) or are in a relationship but have sexual encounters with other individuals than their partners (45%). These patterns are similar across languages.

**TABLE 1:** Sample characteristics (Switzerland, 2020).

	Total (*N* = 802)	English (*n* = 178)	French (*n* = 177)	German (*n* = 447)	*p*
Score (*M*, *SD*)	2.22 (0.58)	2.13 (0.59)	2.32 (0.63)	2.22 (0.63)	0.432
Age (*M*, *SD*)	30.40 (9.95)	39.19 (11.11)	40.73 (10.19)	38.96 (9.32)	0.099
Education, Tertiary^a^ (%)	69.33	73.60	80.79	63.09	<0.001
MSM group^b^, I am currently taking PrEP (%)	88.03	85.96	93.79	86.58	0.020
Sexual relationship (%)					0.283
Single, no sexual contacts	5.99	5.62	5.65	6.26	
Single, with sexual contacts	41.90	38.20	45.76	41.83	
Exclusive relationship	6.89	11.24	5.65	5.59	
Non-exclusive relationship	45.26	44.94	42.94	46.31	

Notes. M, mean; SD, Standard Deviation.

^a^ Tertiary education includes: Short-cycle tertiary education [e.g., HNC/HND (UK)], Bachelor’s or equivalent level, Master’s or equivalent level, and Doctoral or equivalent level.

^b^ MSM group: baseline = I took PrEP before.

*p*-column: Tests for significant differences between language groups using Fisher’s exact test and iterative proportional fitting.

The sample’s average score of the adapted HPSS was 2.22 (*SD* = 0.58), indicating an overall moderate level of PrEP stigma. The French sample reports the highest—still moderate—PrEP stigma, with an average score of 2.32 (*SD* = 0.63). More information about the scale and item distribution (skewness, kurtosis, floor, and ceiling effects) is available in the Supplementary Table SA2 and Supplementary Figure SA2.

### Validity

KMO and Bartlett’s tests for sphericity measures indicate that the data are suitable for factor analysis (*KMO* = 0.71, $\chi^{2}$ = 1758.34, *df* = 78, *p* < 0.001). The scree plot of the eigenvalues evidenced a five-factor solution (see Supplementary Figure SA1), but a four-factor model was retained due to poor factor loadings on the fifth factor.

Table 2 shows the results from the EFA. After oblique rotation, each item loaded on the evidenced four-factor solution, labeled: 1) “Negative social consequences,” 2) “Feeling socially pressured,” 3) “External support,” and 4) “Self-support.” Items 4 and 7 were dropped due to factor loadings below 0.3. The factor structure was confirmed with *CFI* = 0.983, *TLI* = 0.975, and *RMSEA* = 0.047 and *SRMR* = 0.055, indicating that the adapted HPSS is a suitable tool to assess experienced stigma regarding one’s PrEP use within the MSM population.

**TABLE 2:** Retained factor structure of the adapted HIV Pre-exposure Prophylaxis Stigma Scale (HPSS) after Exploratory Factor Analysis (EFA) and correlation matrix among retained factors (Switzerland, 2020).

Factors Items		*M*	*SD*	Factors				Uniqueness
				1	2	3	4	
**1. Negative social consequences**								
3	I experience negative judgement because I take PrEP	1.67	0.99	0.48				0.55
5	I am seen by others as promiscuous (colloquially “slutty”)	2.67	1.26	0.67				0.61
8	People I have sex with who know that I have been taking PrEP before, think that I would like to have condomless sex with others	3.74	1.23	0.40				0.80
10	I experience verbal harassment because I take PrEP	1.44	0.82	0.58				0.67
**2. Feeling socially pressured**								
1	I feel ashamed to take my PrEP pills in front of others	1.78	1.16		0.80			0.37
2	I keep my PrEP pills hidden	2.01	1.35		0.79			0.36
**3. External support**								
12	Family members who know that I am taking PrEP think less of me	2.49	1.40			0.53		0.67
13	Friends who know that I am taking PrEP think less of me	1.87	1.18			0.61		0.65
**4. Self-support^a^**								
6	I receive praise for being responsible by taking PrEP	2.87	1.23				0.51	0.69
9	I feel proud to take PrEP	2.12	1.25				0.64	0.57
11	By taking PrEP, I am doing something for my health	1.89	1.23				0.48	0.77
Correlation among factors								
	Factor 1			—				
	Factor 2			0.52***	—			
	Factor 3			0.70***	0.60***	—		
	Factor 4			−0.08**	−0.22***	−0.22***	—	

Notes. M, mean; SD, Standard Deviation. For analysis, the sample was randomly split in two groups. Sample 1 (*n* = 401) was used to run the EFA while sample 2 (*n* = 401) was used to test the four-factor structure as part of the CFA.

Spearmans Rho correlations: **: *p* > 0.05; ***: *p* > 0.01.

^a^ Reverted scale.

### Measurement Invariance

Table 3 presents the outcomes of the MGCFA analysis (*n* = 802). Initially, three independent baseline models were computed for English, French, and German, all of which exhibited a robust fit. Subsequently, to investigate invariance across language groups, a sequence of three nested models was executed: the configural invariance model (model 1), followed by the metric invariance model (model 2), and finally, the scalar invariance model (model 3).

**TABLE 3:** Measurement invariance of the adapted HIV Pre-exposure Prophylaxis Stigma Scale (HPSS) across three language groups (Switzerland, 2020).

	Fit indices									
	χ^2^	*df*	*p*	*CFI*	*RMSEA*	*SRMR*				
Baseline	154.31	38	<0.001	0.970	0.066	0.058				
English	52.23	38	0.062	0.984	0.048	0.069				
French	54.13	38	0.043	0.980	0.052	0.075				
German	145.09	38	<0.001	0.953	0.085	0.076	**Model Comparisons**			
Model 1	249.57	114	<0.001	0.965	0.071	0.074	Δ’s	*CFI*	*RMSEA*	*SRMR*
Model 2	273.26	128	<0.001	0.963	0.069	0.080	1 vs. 2	−0.002	−0.001	0.006
Model 3	390.31	186	<0.001	0.948	0.068	0.080	2 vs. 3	−0.015	0.001	0.000

Note. *N* = 802. χ^2^, chi-square values; df, degree of freedom; CFI, comparative fit index; RMSEA, root mean square error of approximation; SRMR, standardized root mean square residuals. Change in fit indices is represented by Δ. Model details: Model 1 = Configural invariance, Model 2 = Metric invariance, Model 3 = Scalar invariance.

Model 1 shows a satisfactory fit, confirming configural invariance (*CFI *= 0.965, *RMSEA* = 0.071, *SRMR* = 0.074). When constraining factor loadings to be equal across groups in Model 2, the fit was still reasonable (*CFI* = 0.963, *RMSEA* = 0.069, *SRMR* = 0.080), and changes in fit indices indicate metric invariance (Δ*CFI* = −0.002, Δ*RMSEA* = −0.001, Δ*SRMR* = 0.006). When restricting intercepts to be equal in Model 3, the fit was still satisfactory (*CFI* = 0.948, *RSMEA* = 0.068, *SRMR* = 0.080), and changes in model fit indicated scalar invariance (*ΔCFI* = −0.015, *ΔRMSEA* = 0.001, *ΔSRMR* = 0.000). Therefore, the HPSS-EN/FR/DE is a suitable tool to measure experienced stigma across the three languages.

### Reliability

Cronbach’s alpha and McDonald’s omega were calculated for the overall scale and for each subgroup, i.e., scale factors and language groups. Concerning Cronbach’s alpha, the overall reliability is α = 0.68 and ranges from α = 0.60 (Factor 3) to α = 0.83 (Factors 1 and 2). For McDonald’s omega, the overall reliability is ω = 0.70 and ranges from ω = 0.60 (Factor 4) to ω = 0.83 (Factor 2). Supplementary Table SA3 depicts results of the reliability analysis for the overall sample and per language.

## Discussion

The primary objective of this study was to extend the utility of the HIV Pre-Exposure Prophylaxis Stigma Scale (HPSS), originally formulated and validated by Siegler et al. [18]. Our extension targeted two critical dimensions. Firstly, by translating the scale into French and German, we aimed to facilitate application within a broader linguistic and cultural spectrum, thus enhancing cross-contextual relevance. Secondly, the initial scale’s validation predominantly pertained to non-PrEP users lacking current or prior experience with stigma. Consequently, our study introduces a distinctive contribution by employing it within a sample of (prior) PrEP users. Given the global imperative of HIV prevention, the necessity to equip research and implementation with a robust assessment tool for PrEP-related stigma becomes paramount. This form of stigma constitutes a pivotal impediment influencing medication uptake, adherence, and discontinuation among the MSM community [9]. While the initial scale demonstrated promise within specific contexts, it remained imperative to establish its reliability and validity in more diverse settings, accommodating distinct linguistic and cultural backgrounds.

In our analysis, the construct validity of the instrument was robustly supported through EFA and CFA, applied to separate halves of the sample, revealing a consistent four-factor structure. These factors—1) “Negative social consequences,” 2) “Feeling socially pressured,” 3) “Self-support,” and 4) “External support”—were clearly delineated, with each item demonstrating strong factor loadings, underscoring their relevance to the overall assessment of PrEP-related stigma. Item 8 emerged as an outlier due to its unique loading pattern, prompting a reevaluation of its fit within the instrument. Despite its distinctiveness, we opted to retain this item, as its exclusion did not significantly enhance model fit statistics, nor did it detrimentally affect the scale’s construct validity.

In evaluating the reliability of the HPSS, our findings suggest fair to good reliability across the aggregate data and within the three linguistic subsets. Internal consistency, as measured by Cronbach’s alpha (α), showed variations from the original study, with values ranging from 0.60 to 0.83. In our view, this deviation reflects the complex nature of measuring stigma across different populations, notably among our MSM sample of *current and former PrEP users*, compared to the initial study of the HPSS on MSM *without PrEP experience*. Such differences could contribute to the observed variability in reliability metrics. For McDonald’s omega (ω), we found values between 0.60 and 0.83. Considering both alpha and omega results alongside the outcomes of the EFA and CFA, the scale, despite areas identified for potential refinement, remains a robust and suitable instrument for assessing PrEP-related stigma [31, 32]. Moving forward, the insights gained from this validation process highlight the importance of continuous scale evaluation and refinement to ensure its relevance and efficacy in capturing the multifaceted nature of stigma experienced by PrEP users.

Even though our study effectively employed the HPSS within a cohort of (former) PrEP adopters it remains unclear, whether the findings pertaining to *experienced* stigma align and are comparable to those of a cohort of non-adopters, which encompasses *anticipated* stigma. Crucial factors pertinent to both these manifestations of stigma may exert significant influence when evaluating and contrasting the two constructs. Comparable investigations in different domains, such as problematic gambling [33], have already grappled with this issue. Consequently, a more robust foundation of both theoretical and empirical research is imperative to establish the necessity of integrating supplementary dimensions while assessing diverse sub-populations within the prism of PrEP utilization.

On a related note, another pathway for future studies pertains to the scale’s application beyond the confines of high-income European countries. Recognizing the imperative to combat PrEP-related stigma across a myriad of linguistic and cultural contexts, this research serves as a foundational step towards such a global endeavor. By adapting the HPSS to French and German, we’ve initiated the process of making this instrument accessible in regions with significant Francophone populations, including various African countries. Additionally, exploring the utility of large language models for scale translation presents an innovative approach to further enhance its accessibility and applicability globally. Future research should continue this trajectory, focusing on the validation and reliability of the scale in different settings to ensure its global utility. Such efforts are pivotal for developing a nuanced understanding of PrEP-related stigma across cultural boundaries, which is essential for crafting effective public health interventions and policies to improve PrEP uptake and adherence worldwide.

The sampling strategy and participant recruitment process in this study warrant careful consideration, especially given the multicultural context, the sensitive nature of the topic, and the specific characteristics of the population under investigation. We employed snowball sampling to navigate these complexities, aiming to leverage the social networks of current PrEP users and maximize our sample size. While this approach facilitated access to a population that might otherwise be difficult to reach, it also led to a sample that is particularly informed by current users and those with a higher level of education. This approach, while enabling us to gather rich insights from a variety of cultural backgrounds, necessitates careful interpretation of our findings due to the sample’s lack of representativeness. The existing imbalance also extends to variations in experience with PrEP and encompasses disparities in sample sizes across different countries. While we have accounted for these considerations in our analyses and model estimations, forthcoming research must corroborate these findings to determine their generalizability.

Relatedly, an important limitation of this study is the specific context of data collection during the COVID-19 pandemic (December 2020 to April 2021), a period marked by significant changes in the sexual behavior and PrEP use among MSM. Studies have documented decreased sexual encounters and PrEP utilization among MSM during the pandemic [34, 35]. Although the applied survey inquired about general and past experiences with PrEP-related stigma, the pandemic’s impact on respondents’ perceptions and experiences cannot be entirely excluded. However, given that this study’s primary aim was to validate a measurement instrument for assessing PrEP-related stigma, the influence of these temporary behavioral changes on the scale’s efficacy and relevance is limited.

From a practical public health perspective, the combined outcomes suggest that the experienced stigma level within the sample (across all countries) tends to range from low to moderate. However, this aggregated assessment disguises a significant reality: there are individuals within the sample who report heightened experiences of PrEP-related stigma (see Supplementary Table SA2 and Supplementary Figure SA2). This revelation underscores the pressing need for public health initiatives to persistently combat PrEP stigma. Such efforts hold the potential to enhance the uptake of PrEP and, more crucially, bolster adherence and the sustained use of this preventive medication. The requisite measures include a spectrum of actions, from cultivating awareness through education and information dissemination to mitigating stigma via suitable campaigns. Moreover, creating supportive and non-discriminatory environments across healthcare settings, workplaces, and social circles emerges as a paramount avenue to address this critical issue.

In conclusion, this study verified the reliability and validity of a scale assessing experienced stigma of PrEP use, namely, the HPSS-EN/FR/DE. In extending the initial work of Siegler et al. [18], this broadens the possibilities to apply the scale for (a) a broader target group, i.e., people with ongoing or past use of PrEP, and (b) other cultural contexts, i.e., languages (German and French). Overall, the results underline the reliability and validity of the instrument for the investigated population.

By modifying and conducting rigorous testing of the HPSS, our research enables a more comprehensive examination of a significant obstacle in PrEP uptake and adherence. This effort takes on added importance as it allows for the assessment of stigma levels across various populations and cultural contexts. Consequently, this work provides valuable insights for public health experts to consider tailored interventions that empower individuals to make informed decisions about their sexual health.
