# Supplementary material for: The Pre-Exposure Prophylaxis Stigma Scale: Measurement Properties of an Adaptation in German and French
Source: Int J Public Health. 2024 Apr 29;69:1606658. doi: 10.3389/ijph.2024.1606658 (PMC11089210; doi:10.3389/ijph.2024.1606658)

Supplementary Materials

Table A1. *HPSS-EN/FR/DE 13-items (Switzerland, 2020)*

			1	2	3	4	5
**Item**	**Adopters**	Question	**Strongly agree**	**Agree**	**Neither agree nor disagree**	**Disagree**	**Strongly disagree**
1	Former	I felt ashamed to take PrEP pills in front of others.<br>*Es war mir peinlich, PrEP vor anderen einzunehmen.*<br>*J'avais honte de prendre la PrEP devant les autres.*					
	Current	I feel ashamed to take my PrEP pills in front of others.<br>*Es ist mir peinlich PrEP vor anderen einzunehmen.*<br>*J'ai honte de prendre des comprimés de PrEP devant les autres.*					
2	Former	I kept my PrEP pills hidden.<br>*Ich habe meine PrEP Tabletten vor anderen versteckt.*<br>*Je gardais mes comprimés de PrEP cachés.*					
	Current	I keep my PrEP pills hidden.<br>*Ich verstecke meine PrEP Tabletten.*<br>*Je cache mes comprimés de PrEP.*					
3	Former	I experienced negative judgement because I took PrEP.<br>*Andere hatten eine schlechtere Meinung von mir, weil ich mal PrEP eingenommen habe.*<br>*Ich wurde dafür verurteilt, weil ich PrEP mal eingenommen habe.*<br>*J'ai subi un jugement négatif parce que je prenais la PrEP.*					
	Current	I experience negative judgement because I take PrEP.<br>*Ich werde dafür verurteilt, weil ich PrEP einnehme.*<br>*Je subis un jugement négatif parce que je prends la PrEP.*					

4	Former	Others knowing that I was taking PrEP before was never an obstacle to my sex life.<br><br>*Dass andere wussten, dass ich mal PrEP eingenommen habe, war nie ein Hindernis für mein Sexualleben.*<br><br>*Le fait que d'autres sachent que je prenais la PrEP auparavant n'a jamais été un obstacle à ma vie sexuelle.*					
	Current	Others knowing that I am taking PrEP has never been/is not an obstacle to my sex life.<br><br>*Dass andere wissen, dass ich PrEP einnehme, war noch nie ein Hindernis für mein Sexualleben.*<br><br>*Le fait que d'autres sachent que je prends de la PrEP n'a jamais été/n'est pas un obstacle à ma vie sexuelle.*					
5	Former	I was seen by others as promiscuous (colloquially "slutty").<br><br>*Ich wurde von anderen als promiskuitiv (umgangssprachlich auch "schlampig") betrachtet.*<br><br>*J'étais vu·e par les autres comme étant une personne dévergondée.*					
	Current	I am seen by others as promiscuous (colloquially "slutty").<br><br>*Ich werde von anderen als promiskuitiv (umgangssprachlich auch "schlampig") betrachtet.*<br><br>*Je suis vu·e par les autres comme étant une personne dévergondée.*					

6	Former	I received praise for being responsible when I was taking PrEP.<br><br>*Als ich PrEP genommen habe, wurde ich für mein Verantwortungsbewusstsein gelobt.*<br><br>*J'ai été félicité(e) de mon attitude responsable lorsque je prenais la PrEP.*	□	□	□	□	□
	Current	I receive praise for being responsible by taking PrEP.<br><br>*Ich werde durch die PrEP-Einnahme für mein Verantwortungsbewusstsein gelobt.*<br><br>*Je suis félicité·e de mon attitude responsable en prenant la PrEP.*					
7	Former	I am treated differently by my doctor and/or his personnel who know that I have been taking PrEP before.<br><br>*Ich werde von meinem Arzt und/oder den Angestellten bei meinem Arzt, die wissen, dass ich mal PrEP genommen habe, anders behandelt.*<br><br>*Je suis traité·e différemment par mon médecin et/ou son personnel qui sait/savent que je prenais la PrEP auparavant.*		□	□	□	□
	Current	I am treated differently by my doctor and/or his personnel who know that I am taking PrEP.<br><br>*Ich werde von meinem Arzt und/oder den Angestellten bei meinem Arzt, die wissen, dass ich PrEP nehme, anders behandelt.*<br><br>*Je suis traité·e différemment par mon médecin et/ou son personnel qui sait/savent que je prends la PrEP.*					

8	Former	People I have sex with who know that I have been taking PrEP before, think that I would like to have condomless sex with others.<br><br>*Personen mit denen ich Sex habe und wissen dass ich mal PrEP genommen habe denken, dass ich gerne kondomlosen Geschlechtsverkehr mit anderen hätte.*<br><br>*Les personnes avec lesquelles j'ai des relations sexuelles qui savent que je prenais la PrEP auparavant, pensent que j'aimerais avoir des relations sexuelles sans préservatif avec d'autres personnes.*	☐	☐	☐	☐	☐
	Current	People I have sex with who know that I am taking PrEP think that I would like to have condomless sex with others.<br><br>*Personen mit denen ich Sex habe und wissen dass ich PrEP nehme denken, dass ich gerne kodomlosen Geschlechtsverkehr mit anderen hätte.*<br><br>*Les personnes avec lesquelles j'ai des relations sexuelles qui savent que je prends la PrEP pensent que j'aimerais avoir des relations sexuelles sans préservatif avec d'autres personnes.*					
9	Former	I felt proud to take PrEP.<br><br>*Ich war stolz PrEP einzunehmen.*<br><br>*J'étais fier·ère de prendre la PrEP.*	☐	☐	☐	☐	☐
	Current	I feel proud to take PrEP.<br><br>*Ich bin stolz PrEP einzunehmen.*<br><br>*Je suis fier·ère de prendre la PrEP.*					
10	Former	I experienced verbal harassment because I took PrEP.<br><br>*Ich wurde verbal angefeindet, weil ich mal PrEP eingenommen habe.*<br><br>*J'ai été victime de harcèlement verbal car je prenais la PrEP.*	☐	☐	☐	☐	☐
	Current	I experience verbal harassment because I take PrEP.<br><br>*Ich werde verbal angefeindet, weil ich PrEP einnehme.*<br><br>*Je suis victime de harcèlement verbal car je prends la PrEP*					

11	Former	By taking PrEP, I am doing something for my health.<br><br>*Durch die Einnahme von PrEP tat ich etwas für meine Gesundheit.*<br><br>*En prenant la PrEP, je faisais quelque chose pour ma santé.*	□	□	□	□	□
	Current	By taking PrEP, I am doing something for my health.<br><br>*Durch die Einnahme von PrEP tue ich etwas für meine Gesundheit.*<br><br>*En prenant la PrEP, je fais quelque chose pour ma santé.*					
12	Former	Family members who know that I have been taking PrEP before think less of me.<br><br>*Familienmitglieder, die wissen, dass ich mal PrEP genommen habe, haben eine schlechtere Meinung von mir.*<br><br>*Les membres de ma famille, qui savent que je prenais la PrEP auparavant, ont une moins bonne opinion de moi.*	□	□	□	□	□
	Current	Family members who know that I am taking PrEP think less of me.<br><br>*Familienmitglieder, die wissen, dass ich PrEP nehme, haben eine schlechtere Meinung von mir.*<br><br>*Les membres de ma famille, qui savent que je prends la PrEP, ont une moins bonne opinion de moi.*					
13	Former	Friends who know that I have been taking PrEP before think less of me.<br><br>*Freunde, die wissen, dass ich mal PrEP genommen habe, haben eine schlechtere Meinung von mir.*<br><br>*Mes amis, qui savent que je prenais la PrEP auparavant, ont une moins bonne opinion de moi.*	□	□	□	□	□
	Current	Friends who know that I am taking PrEP think less of me.<br><br>*Freunde, die wissen, dass ich PrEP nehme, haben eine schlechtere Meinung von mir.*<br><br>*Mes amis qui savent que je prends la PrEP ont une moins bonne opinion de moi.*					

Table A2. *Basic description of the HPSS-EN/FR/DE items (Switzerland, 2020)*

Item	*M*	*SD*	Skewness	Kurtosis	Floor (%)	Ceiling (%)
1	1.78	1.16	1.33	3.64	61.43	3.86
2	2.01	1.35	1.02	2.61	55.86	7.59
3	1.67	0.99	1.41	4.19	61.38	1.66
4*	2.38	1.57	0.67	1.86	41.99	19.06
5	2.67	1.26	0.06	1.84	25.79	6.34
6	2.87	1.23	0.15	2.18	16.04	13.14
7*	1.79	1.14	1.21	3.27	61.80	2.92
8	3.74	1.23	-0.93	2.85	11.01	33.69
9	2.12	1.25	0.89	2.79	43.65	7.60
10	1.44	0.82	2.01	6.58	72.51	0.65
11	1.89	1.23	1.33	3.71	55.17	7.72
12	2.49	1.40	0.40	1.85	36.52	11.55
13	1.87	1.18	1.22	3.45	55.17	4.83
Total	2.22	0.58	0.40	2.93		
HPSS-EN	2.13	0.59	0.53	3.00		
HPSS-FR	2.32	0.63	0.17	2.38		
HPSS-DE	2.22	0.55	0.45	3.29		

Note. This table shows the results of the descriptive analysis for each item, overall and per language. Except for item 4 and 8, no ceiling effect was detected. *M* = Mean, *SD* = Standard Deviation. Floor effect is present for each item except item 8 (at least 15% of respondents answered the lowest score, i.e., 1). Skewness and kurtosis ranges between -0.93 and 2.01 and 1.84 and 6.58, respectively. Items 1, 3, 7, 10, 11, and 13 display a kurtosis coefficient larger than 3, a sign of data distributed with pronounced peaks and long and flat tails.

*items dropped after EFA.

Table A3. *Inter-item correlation and alpha overall and per factor (Switzerland, 2020)*

Factors	Average inter-item correlation	Language			
		Overall (all lang.)	English	French	German
Overall	0.17				
Cronbach’s alpha (α)	-	0.68	0.69	0.69	0.68
McDonald's Omega (ω)	-	0.70	0.70	0.71	0.70
Factor 1: Negative social consequences		0.39			
Cronbach’s alpha (α)	-	0.83	0.60	0.55	0.68
McDonald's Omega (ω)	-	0.65	0.62	0.58	0.69
Factor 2: Feeling socially pressured		0.71			
Cronbach’s alpha (α)	-	0.83	0.82	0.81	0.85
McDonald's Omega (ω)	-	0.83	0.82	0.81	0.85
Factor 3: Self-support		0.33			
Cronbach’s alpha (α)	-	0.60	0.63	0.54	0.60
McDonald's Omega (ω)	-	0.69	0.75	0.71	0.63
Factor 4: External support					0.43
Cronbach’s alpha (α)	-	0.64	0.71	0.66	0.58
McDonald's Omega (ω)	-	0.60	0.63	0.54	0.60

Note. Standardized scores used for computation. Standardized items reported. Factor 2 and 4 consist of 2 items, therefore values can be interpreted as correlation coefficients (*r*)

Figure A1. *Scree plot and parallel analysis (Switzerand, 2020)*

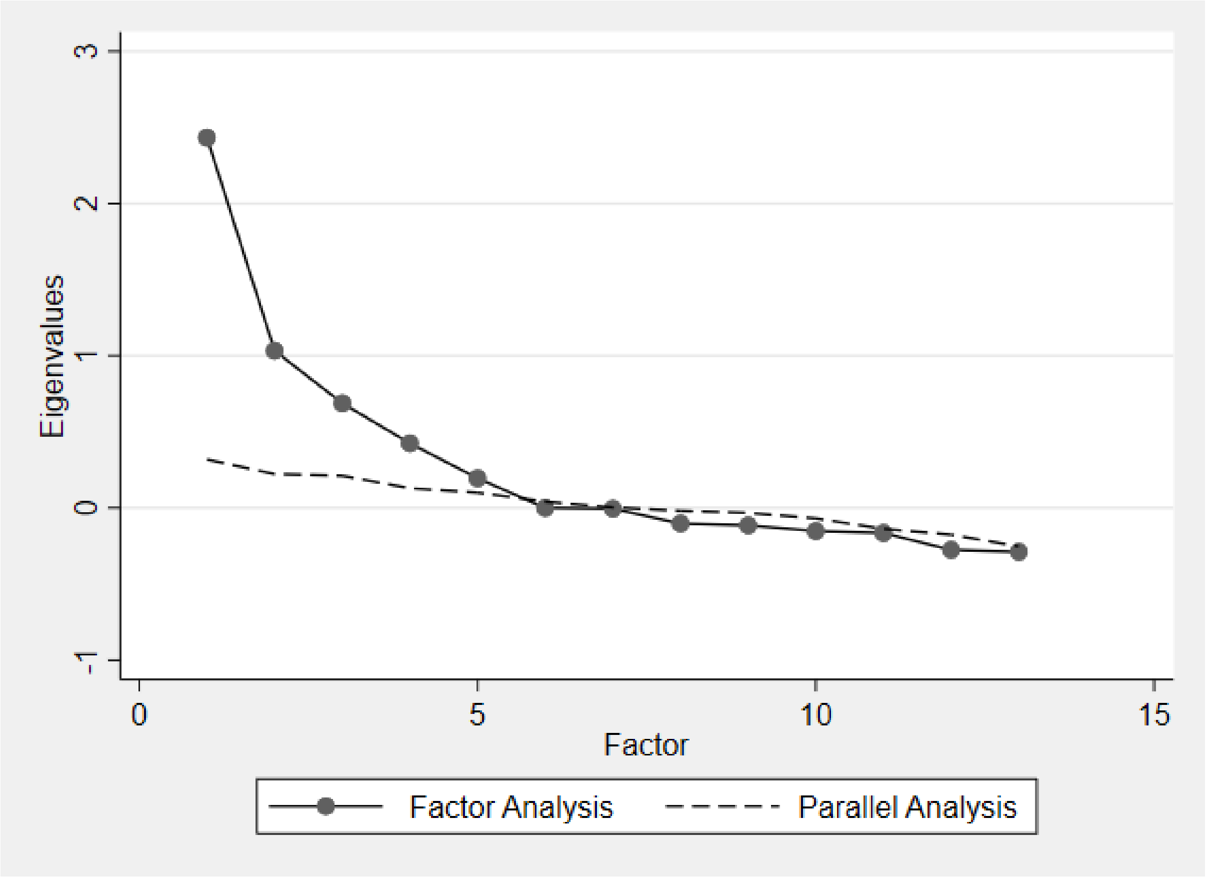

Figure A2. *Distribution of stigma scores for the overall sample (Switzerland, 2020)*

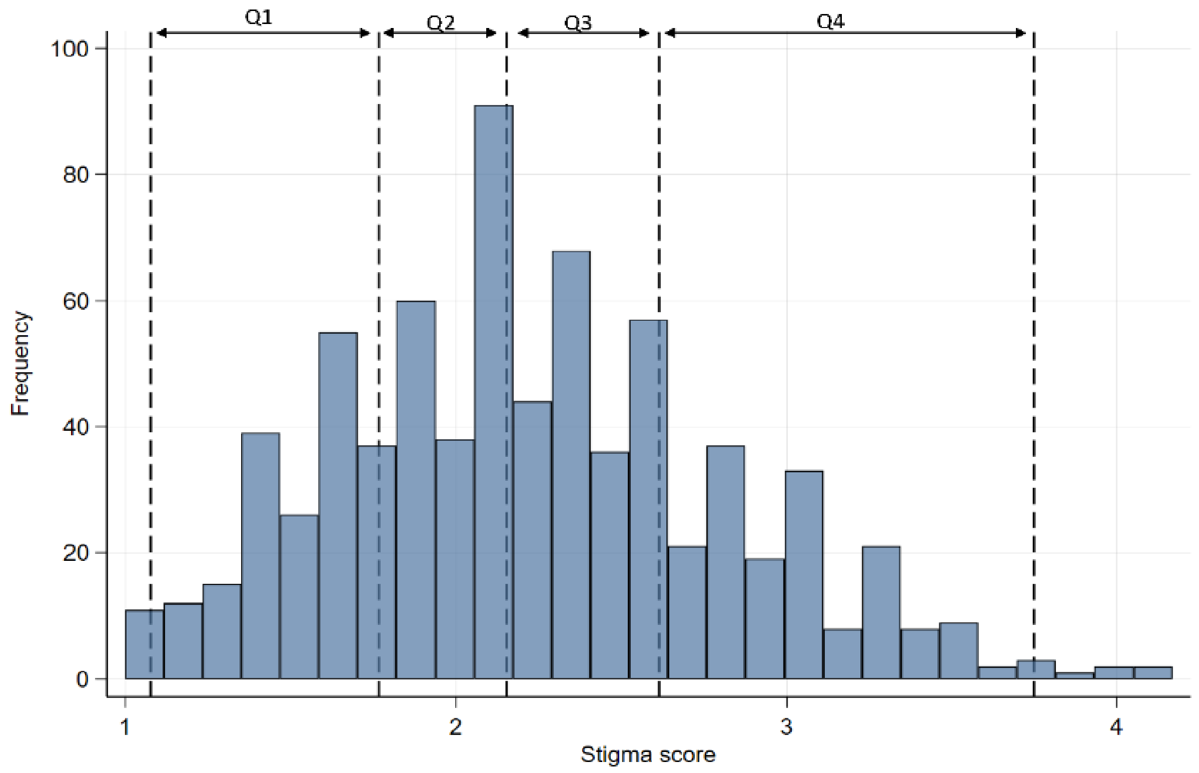

Supplement: Supplementary file 1 [file DataSheet1.zip › Ort_Bardy_Supplement.pdf]
